# Subcellular Dissection of a Simple Neural Circuit: Functional Domains of the Mauthner-Cell During Habituation

**DOI:** 10.3389/fncir.2021.648487

**Published:** 2021-03-22

**Authors:** Dániel Bátora, Áron Zsigmond, István Z. Lőrincz, Gábor Szegvári, Máté Varga, András Málnási-Csizmadia

**Affiliations:** ^1^MTA-ELTE Motor Pharmacology Research Group, Budapest, Hungary; ^2^Printnet Limited, Budapest, Hungary; ^3^Motorpharma Limited, Budapest, Hungary

**Keywords:** zebrafish, startle, glutamate-release, subcellular domains, sensorimotor integration

## Abstract

Sensorimotor integration is a pivotal feature of the nervous system for ensuring a coordinated motor response to external stimuli. In essence, such neural circuits can optimize behavioral performance based on the saliency of environmental cues. In zebrafish, habituation of the acoustic startle response (ASR) is a simple behavior integrated into the startle command neurons, called the Mauthner cells. Whereas the essential neuronal components that regulate the startle response have been identified, the principles of how this regulation is integrated at the subcellular regions of the Mauthner cell, which in turn modulate the performance of the behavior, is still not well understood. Here, we reveal mechanistically distinct dynamics of excitatory inputs converging onto the lateral dendrite (LD) and axon initial segment (AIS) of the Mauthner cell by *in vivo* imaging glutamate release using iGluSnFR, an ultrafast glutamate sensing fluorescent reporter. We find that modulation of glutamate release is dependent on NMDA receptor activity exclusively at the AIS, which is responsible for setting the sensitivity of the startle reflex and inducing a depression of synaptic activity during habituation. In contrast, glutamate-release at the LD is not regulated by NMDA receptors and serves as a baseline component of Mauthner cell activation. Finally, using *in vivo* calcium imaging at the feed-forward interneuron population component of the startle circuit, we reveal that these cells indeed play pivotal roles in both setting the startle threshold and habituation by modulating the AIS of the Mauthner cell. These results indicate that a command neuron may have several functionally distinct regions to regulate complex aspects of behavior.

## Introduction

Special neural circuits enable the animal to effectively distinguish sensory information based on saliency before making a behavioral decision. One pivotal example of sensorimotor integration is habituation, a simple form of non-associative learning, manifested as the progressive decline of a particular behavioral response to repeated sensory stimulation (Thompson and Spencer, 1966; Giles and Rankin, 2009). While the neural mechanisms underlying habituation remain largely unclear on the circuit level, deficits in this process are prevalent in neurological disorders such as schizophrenia (Akdag et al., 2003; Williams et al., 2013), Parkinson’s disease (Chen et al., 2016) and autism spectrum disorder (Constantin et al., 2020).

Many studies have provided insights into the possible cellular mechanisms behind the progressive attenuation of behavioral responses during habituation (Krasne and Bryan, 1973; Stopfer et al., 1996; Weber et al., 2002; Ezzeddine and Glanzman, 2003; Bristol and Carew, 2005). The model of homosynaptic depression, for example, suggests that it is the depletion of the pool of presynaptic neurotransmitter vesicles that leads to the attenuation of a monosynaptic circuit and drives habituation. In other models, the gradual potentiation of a feedforward inhibitory neuron population is presented as the source of the modulation of the excitability of the neuron controlling the behavioral output.

The similarity between results obtained from genetic and neuropharmacological experiments modulating habituation in invertebrate and vertebrate models suggests that the key mechanisms underlying this behavior are evolutionarily conserved (Bickel et al., 2008; Giles and Rankin, 2009; Wolman et al., 2011, 2015; Klamer et al., 2014). In this study, we chose the habituation of the acoustic startle response (ASR) of zebrafish larvae as a model due to the relative simplicity of the neural circuits regulating the behavioral output and the accessibility of genetic and pharmacological tools that can be used to manipulate and monitor neural activity *in vivo*.

**Figure 1:**
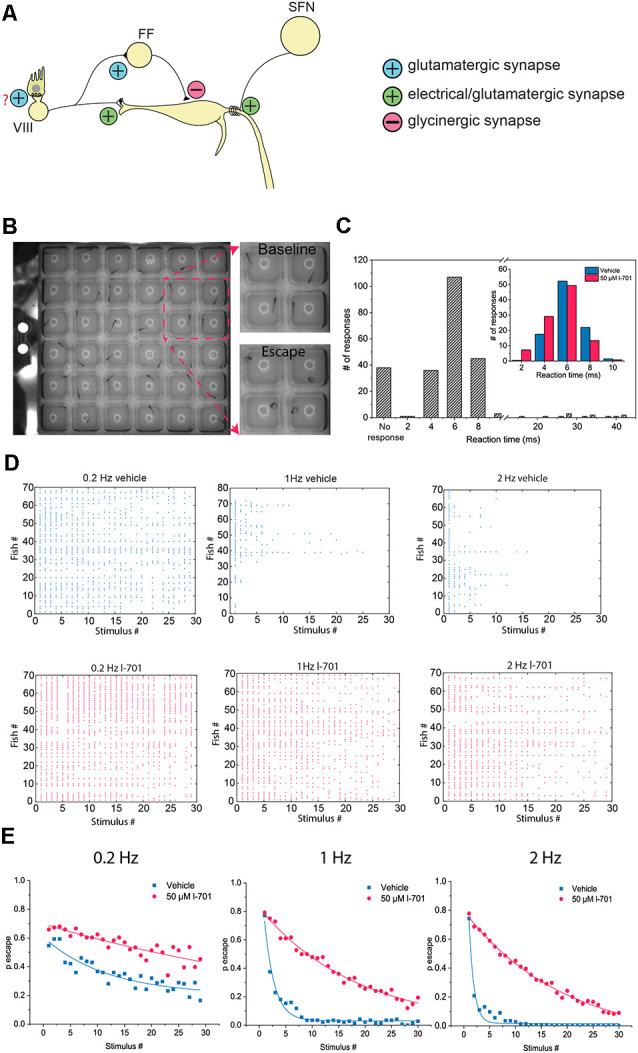
Characteristics of the M-cell mediated escape response, and its habituation. **(A)** Schematic representation of the startle circuit located in the hindbrain. The M-cell, a command neuron responsible for eliciting a startle response receive two excitatory inputs: VIII nerve inputs at the distal lateral dendrite (LD), and inputs from spiral fiber neurons (SFN) at the axon initial segment (AIS) *via* a feedforward excitatory circuit. Additionally, the M-cell is innervated by a glycinergic feedforward inhibitory circuit converging on the soma of the M-cell. **(B)** Representative image of an M-cell mediated escape response. Fish were placed in individual wells on a 6 × 6 custom plate with a speaker underneath delivering acoustic-vibrational stimuli. A high-speed camera set up above the plate was used to record the behavior. **(C)** Histogram showing the latency of observed escape responses. M-cell mediated escapes, often referred to as short-latency C-bends (SLC) are elicited approximately 2–12 ms after stimulus onset. Longer latency escape responses are mediated by neural circuits other than the M-cell. With our stimulus-delivery approach, the most frequently observed escapes happened 4–8 ms after the stimulus, consistent with the time window the M-cell operates in. Longer latency escapes were rarely observed. Latencies were manually calculated. Two-hundred and forty-three responses of 81 fish were measured. Inset: Fish treated with either 50 μM l-701 or vehicle show no significant difference in their distribution of response latency values ( *n* = 95, 100 responses from 19, 20 fish, *p* = 0.09, Kolmogorov–Smirnov Test). **(D)** Repetitive acoustic-vibrational stimulation using the 6 × 6 plate causes a progressive decline in escape responses. This decline is dependent on the frequency of the stimulation, with higher frequencies causing more rapid habituation. Ninety-min incubation in 50 μM l-701 greatly disrupts the habituation of the escape response (vehicle: *n* = 70, 80, and 70 for 0.2 Hz, 1 Hz, and 2 Hz, respectively; l-701: *n* = 70, 70 and 70 for 0.2 Hz, 1 Hz and 2 Hz, respectively). **(E)** Plotting the probability of an escape response at each time point during the stimulation based on averages from all fish, exponential fit used.

In larval zebrafish abrupt acoustic-vibrational stimuli trigger a swift escape behavior, the ASR, also termed as the “C-bend” after the shape of the fish’s unilaterally contracted muscles. Distinct cell populations of homologous hindbrain circuits drive startle responses of various latencies depending on the sensory origin of the stimulus (Kohashi and Oda, 2008). The short-latency (2–12 ms) ASR is mediated by the activity of a pair of giant hindbrain neurons, called the Mauthner cells (M-cells; Hale et al., 2016). The M-cells serve as an interface of sensorimotor integration, responsible for the coordination of motor responses to multisensory synaptic activities, mainly from vestibular and lateral line afferents. The role of the M-cells and adequate regulation of M-cell activity in reflex initiation is crucial, as even a single action potential in one of the cells is sufficient to trigger the motor response (Nissanov et al., 1990). Interneurons responsible for the regulation of the motor output are clustered into functional units along rhombomeres in the dorso-ventral dimensions of the hindbrain (Koyama et al., 2011). The M-cells receive input from two feedforward interneuron circuits: a glycinergic input converging onto the M-cell soma located in rhombomere 4 and the recently discovered glutamatergic spiral fiber neurons (SFN) ending at the axon initial segment (AIS) of the M-cell located at rhombomere 3 (Lacoste et al., 2015; Figure 1A).

The habituation-related role of the main afferents endings on the M-cell has been investigated thoroughly in the last few decades. Patch-clamp studies emphasized the possible contribution of homosynaptic depression at afferent connections of the vestibulocochlear cranial nerve, as repetitive potentiation of the axons resulted in a progressive decline in synaptic transmission to the M-cell lateral dendrite (LD; Pereda et al., 2004). *In vivo* calcium imaging revealed a decrease in M-cell excitability during habituation, which along with the pharmacological blockade of NMDA and glycine receptors suggested that both potentiated activity of feedforward inhibitory neurons and depression of auditory (VIII) nerve afferents lead to the decline in behavioral output (Marsden and Granato, 2015). Other articles focusing on the role of spiral fiber neurons have concluded that the activity of these cells is crucial for eliciting a “C-bend” response and setting the startle threshold, yet the mechanistic details of their contribution to habituation have not been investigated (Lacoste et al., 2015; Marsden et al., 2018). While all of these studies provided some insight into the possible mechanisms that drive habituation, it is still unresolved how the individual activities of the converging inputs shape this behavior on a subcellular level. In this paper, we sought to investigate how activities of excitatory inputs converging on the M-cell are modulated on a subcellular level during habituation. To achieve this, we express the fluorescent glutamate reporter iGluSnFR in the M-cell, allowing simultaneous measurement of fluorescence changes in multiple subcellular regions during habituation of the startle response. We also investigate if these activities can be perturbed when habituation is impaired by NMDA antagonists, as these receptors are thought to play a fundamental part in the adaptation process (Roberts et al., 2011; Marsden and Granato, 2015).

## Materials and Methods

### Generation and Husbandry of Zebrafish Lines

*Tg(hspGFF62A:Gal4)* fish were kindly provided by Koichi Kawakami (Asakawa et al., 2008). *Tg(10xUAS:iGLuSnFR)* were provided by Sabine Renninger. *Tg(-6.7Tru.Hcrtr2:Gal4-VP16;UAS:GCaMP5) (pSAM)* fish were kindly provided by Alex Schier and David Schoppik. The transgenic fish lines, as well as the wild type AB and ekwill lines, were maintained in the animal facility of ELTE Eötvös Loránd University according to standard protocols (Westerfield, 2007; Aleström et al., 2020). Fish were group-housed and maintained in a standard 14/10 h light/dark cycle. Feeding of larvae started at 5 days post fertilization (dpf) using a combination of commercially available dry food (100–200 μm Zebrafeed, Sparos) and paramecium. This regimen was used until 15 dpf. After that, juvenile fish were fed using dry food with gradually increasing particle size (200–400 μm Zebrafeed) in combination with fresh brine shrimp hatched in the facility. Adult fish from 30 dpf were fed with dry food (Small Granule, Special Diets Services, product code: 824876) combined with brine shrimp. Water quality was controlled constantly by a Stand-Alone (Tecniplast) system, parameters set to: pH 8.0, 500 μS, 28.5°C. Fish expressing the *iGluSnFR* construct in the Mauthner cells was created by crossbreeding *Tg(hspGFF62A:Gal4)* and *Tg(10xUAS:iGluSnFr)* strains. Experimental subjects were larvae aged 5–7 dpf kept in E3 medium (5 mM NaCl, 0.33 mM MgCl_2_, 0.33 mM CaCl_2_, 0.17 mM KCl) in a dark incubator set to 28.5°C. Animals were terminated on ice after experiments. All protocols employed in our study were approved by the Hungarian National Food Chain Safety Office.

### Behavioral Analysis, Drug Treatment

At the age of 5–7 dpf, wild-type zebrafish were placed individually into a custom-built 6 × 6-welled plastic plate with the following parameters: well thickness = 0.7 mm, well depth = 5.1 mm, well size = 9 × 9 mm. A small speaker delivering acoustic-vibrational stimuli was placed underneath the plastic plate (Visaton WS 13 E). A brief, 5 ms long sine wave was used at 91.7 dB as the acoustic-vibrational stimulus. Behavioral responses were recorded by a high-speed camera (xiQ USB3 vision) at 500 fps using custom-made software (Printnet Limited) Evaluation of the behavioral recordings was done manually. The latencies of escape responses were determined by flashing a 780 nm LED placed in front of the setup simultaneously with the start of the acoustic-vibrational stimulus delivered by the speaker every time. The flash of the LED was not visible to the fish. All drugs used were bath-applied, administered in 50 μM concentrations, which were diluted from 50 mM 100% DMSO stocks in E3 medium to keep DMSO levels as low as possible. Vehicle controls contained 0.1% DMSO in E3 medium. Each well was filled with 200 μl of the given solution.

### Head-Mounted Behavioral Assay Combined With Glutamate Imaging

All fish subjected to two-photon imaging were treated with 200 μM phenylthiourea (PTU) at 24 h post-fertilization (hpf) to reduce pigmentation. For glutamate-imaging, fish were first anesthetized by incubation in an E3 medium containing 168 mg/l MS-222 (Tricaine) for 2 mins and then were placed into a 35 mm imaging dish (MolBiTec, Imaging dish 1.5). Fish were immobilized in the imaging dish using 2% low melting point agarose (Sigma–Aldrich), then their tails were freed. The anesthetic solution was replaced by washing three times with 1 ml of E3 solution. After anesthesia, fish recovered for 20 min before imaging, and the startle reflex was tested by tactile stimulation of the tail. The imaging dish was fixed in a custom-built plate with a small speaker underneath for acoustic-vibrational stimulus delivery (Visaton FRWS5). The thickness of the plate was chosen to match the thickness of the plate used for behavioral imaging after fixing the imaging dish. To record behavioral responses during two-photon imaging, a high-speed camera (xiQ USB3 vision) was placed at a 45° angle, and the imaging dish was irradiated by two lasers at 780 nm. A bandpass filter was placed in front of the camera to cut out the two-photon laser’s light (Comar 780 IU 25). Imaging of glutamate release was carried out using line-scanning at 250 Hz, 920 nm with a two-photon microscope (Femtonics 2D). Fluorescent responses from the AIS regions were measured 10 μm medially from the soma, while the LD region was captured at the intersection of two lateral branches, which were in the same focal plane as the AIS. Fluorescent responses at the two branches and their intersection were identical, consistent with IHC staining of the large myelinated club endings of the VIII cranial nerve ending on the lateral dendrite. Changes in fluorescence (dF/F0) were calculated using the measuring software of the two-photon microscope (MES, Femtonics 2D), the baseline of a given ROI was determined as the fluorescence value before stimulation. To smoothen the recordings, the line-scans were Gaussian-filtered with two times the standard deviation using the MES software. For single stimulus experiments, dF/F0 values were normalized to the values corresponding to acoustic stimulation at 91.7 dB. For repetitive stimulus experiments, values were normalized to the average of the responses to the first five stimuli. For habituation experiments, 60 stimuli at an interstimulus interval of 1 s 500 ms, and 250 ms were used corresponding to 1, 2, and 4 Hz stimulations, respectively.

### Calcium Imaging of Spiral Fiber Neurons With GCaMP5

Doubly homozygous Tg*(-6.7Tru.Hcrtr2:Gal4-VP16; UAS:GCaMP5*) (*pSAM*) fish were selected based on high GCaMP expression in the notochord cells at 24–36 hpf. Locating the spiral fiber neurons was achieved by focusing on the decussation of the M-cell axons, which is visible under transmitted light. At this plane, the axon cap is apparent. Panning rostral from the axon cap revealed the medial longitudinal fasciculus, along which the somata of the spiral fiber neurons are located. Spiral fiber neurons appeared in two clusters: one rostral and one caudal, 100 μm rostral from the axon cap. Calcium responses were recorded from every cell using line-scans at 20 Hz. Most of the time, only one cell out of the two clusters responded to stimulation. Fluorescence change calculation and normalization were done in the same manner as for the glutamate release imaging experiments. Cell activity was defined as fluorescence values above the mean +2 times the standard deviation of the recording. Two exponentials were fitted to the extracted trace. Fits with a Mean Squared Error (MSE) of more than 10 or no optimal solution for the fit were discarded from the analysis.

### Motion Correction of iGluSnFR and GCaMP5 Data

To correct for Z motion drift, a Z-stack consisting of 30 slices (2 μm increment) was collected before analysis, and the focal plane for the experiment was determined. After each acoustic stimulation, an image was recorded and cross-correlated with the reference slices. The focal plane was then adjusted for maximum cross-correlation. To compensate for potential motion artifacts at the X-Y plane, a total of three parallel and two perpendicular lines were recorded per region of interest (the length of each line was 30 μm). Recordings with substantial motion artifacts were discarded from the analysis following manual evaluation of the traces. Traces with minimal X-Y drift were corrected using maximal cross-correlation to the mean trace of the first 150 data points. Single glutamate or calcium spikes were extracted from the recording (mean trace +2 times the standard deviation), data were binned by a factor of 5, and two exponentials were fitted to the extracted spike. The error of the fit was evaluated by MSE, and spikes with no solution for the fit, or an MSE greater than 10 were discarded from the analysis.

### Data Analysis

Statistical analysis was done using OriginPro (OriginLab). All data are represented as mean ± standard deviation. Significance was determined using two-sample *t*-tests or one-way ANOVA with *post hoc* tests as applicable (*p < 0.05, **p < 0.01, ***p < 0.001). Normality was tested using the Shapiro-Wilk test. Code for the post-processing of glutamate and calcium traces is available at https://github.com/danielbatora/zebrafish_mauthner_analysis.

## Results

### Characteristics of Habituation of the Acoustic Startle Response and the Modulatory Effects of NMDA Receptor Blockade

To assess the characteristics of behavioral responses of larval zebrafish to repeated acoustic stimulation, we established a behavioral assay using a custom-built 6 × 6 plate with a speaker attached underneath (Figure 1B). First, by measuring the reaction time, we confirmed that around 80 percent of the fish responded within the latency range in which the M-cell network operates (Figure 1C; Liu and Fetcho, 1999). Only a small percentage of the responses had longer latencies which indicated that they were not mediated by the M-cell, but by homologous circuits instead (Kohashi and Oda, 2008). Next, we exposed the fish to repetitive stimuli at various frequencies, which resulted in a sharp, frequency-dependent decline in the probability of eliciting a startle response. As the activation of NMDA receptors is believed to be one of the factors behind the habituation of M-cell mediated responses (Marsden and Granato, 2015), we next investigated if NMDA receptor antagonism disrupted habituation at the frequencies measured. Indeed, treatment with 50 μM glycine-site antagonist l-7, 01, 324 (l-701) for 90 min drastically increased the probability of escape at the frequency range measured (Figures 1D,E). Furthermore, there was no significant difference between the escape reaction times of control and treated animals, which suggests that the observed effect on habituation is likely caused by altering the properties of the M-cell network and not by homologous neural circuits (inset in Figure 1C).

### *In vivo* Imaging of Presynaptic Glutamate Release at Multiple Subcellular Regions by Transgenic Zebrafish Expressing iGluSnFR in the Mauthner-Cells

**Figure 2:**
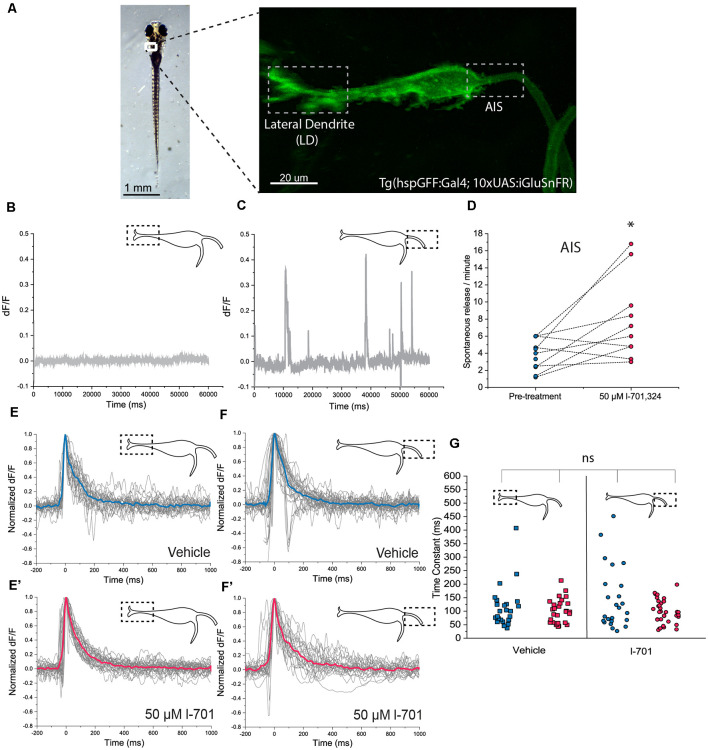
*In vivo* detection of glutamate-release on the M-cell by iGluSnFR. **(A)** Representative image of a transgenic *Tg(hspgGFF62A:Gal4; 10xUAS:iGluSnFR)* fish (6 dpf) in the M-cell region (dorsal view, anterior on top). As previously described, the M-cell receives two major glutamatergic inputs: direct connections from the VIII. nerve ends on the lateral dendrite (left gray box) while inputs coming from a feedforward excitatory network form the axon cap surrounding the AIS of the M-cell (right gray box). **(B,C)** Examples of a 1-min line-scans detecting spontaneous glutamate-release and revealing differences between the LD and the AIS using two-photon imaging. Spontaneous release was completely absent from the LD but was present in an infrequent and unordered fashion at the AIS, with an average of 2–4 releases/minute. **(D)** Administration of l-701 significantly increased the frequency of spontaneous glutamate-release at the AIS (each dot represents an average of three measurements, *n* = 12 fish, *p* = 0.02, paired sample *t*-test). **(E,E’)** The kinetic characteristics of the fluorescent signal after acoustic stimulation indicating glutamate release at the LD in the vehicle (*n* = 5 fish; five repetitions per fish) and 1–701 treated larvae (*n* = 6 fish; five repetitions per fish). **(F,F’)** The kinetic characteristics of the fluorescent signal after acoustic stimulation indicating glutamate release at the AIS in the vehicle (*n* = 5 fish; five repetitions per fish) and 1–701 treated larvae (*n* = 7 fish; five repetitions per fish). **(G)** Kinetic response curves were normalized and compared based on the time constant, which revealed no significant difference between the two regions of interest. Treatment with l-701 did not cause alterations in response dynamics either (five traces per fish, *n* = 5, 6, 5, 7, respectively, *p* = 0.77, one-way ANOVA). *p < 0.05; ns, not significant.

We aimed to functionally dissect the neural circuit responsible for habituation by a novel approach. Previous models focusing on the regulation of M-cell activity emphasized the feedforward glycinergic neurons as being responsible for the attenuation of M-cell mediated actions (Marsden and Granato, 2015). However, no study quantified how the activities of excitatory inputs converging on the M-cell are modulated during behavior. To this end, we set out to investigate the dynamics of activities of glutamatergic afferents during escape responses and habituation. Several glutamatergic components throughout the acoustic startle circuit could regulate the properties of the startle threshold and habituation: the synapses at the hair cells (Obholzer et al., 2008), the lateral dendrite, and spiral fiber neurons (Koyama et al., 2011). The M-cell itself receives excitatory inputs converging on two main subcellular regions: synapses from the VIII cranial nerve ending at the distal LD and input coming from spiral fiber neurons at the proximal AIS. We aimed to measure activities from LD and AIS simultaneously. To achieve this *in vivo*, we used a transgenic zebrafish line expressing the glutamate-release indicator iGluSnFR in the M-cell (Figure 2A; Marvin et al., 2013).

First, we assessed the baseline properties of the two regions of interest. Spontaneous glutamate release was completely absent at the LD region (Figure 2B), however, it was observed at the AIS region with approximately four release events/minute frequency (Figure 2C). Administration of 50 μM of the NMDA receptor blocker l-701 doubled spontaneous glutamate release events at the AIS (Figure 2D), while the properties of the LD remained unaltered (data are not shown). Next, we measured glutamate release in response to acoustic stimulation at both regions of interest using a built-in speaker system underneath the imaging-dish. Acoustic stimulation at 91.7 dB caused fluorescence increase throughout the M-cell, which was most prominent at the LD, and the AIS, where excitatory inputs converge. We first compared the kinetics of release-events at the two sites, and found no significant difference in the latency, with decay times of around 100 ms (Figures 2E–F’). Fifty μM l-701 did not alter the latency of glutamate-release at these regions, as indicated by a scatter-plot comparing time constant values of all conditions (Figure 2G).

**Figure 3:**
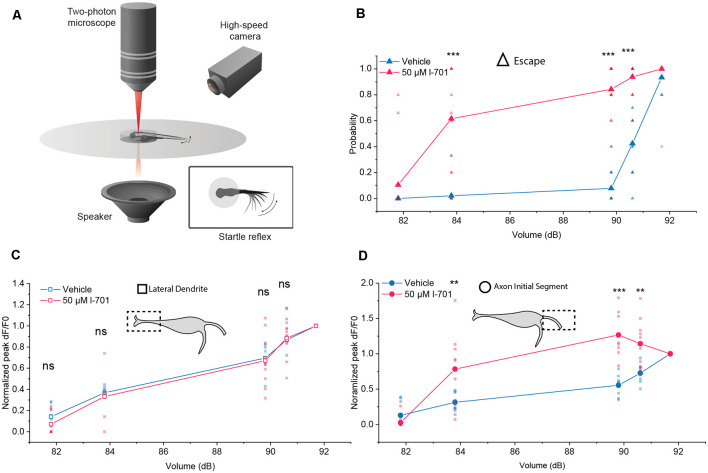
Differential properties of the LD and the AIS during setting the startle threshold. **(A)** Schematic representation of the setup used to monitor M-cell activity during escape response. **(B)** Probability of eliciting a startle response in the presence or absence of l-701 as a function of acoustic volume (dB) recorded simultaneously to glutamate-imaging. 50 μM l-701 significantly elevated the probability of startle responses at volumes larger than 83.8 dB (*n* = *p* < 0.001, two-sample *t*-test). No significant difference was observed at 81.8 dB. **(C)** Glutamate-release at the LD in the presence or absence of l-701 as a function of acoustic volume (dB). We used five different volumes, ranging from 81.8 dB to 91.7 dB, selected in such a way that three of these were well below the threshold level for eliciting an escape response, one near and one above it. Each fish received a series of five acoustic stimuli at each intensity. Fluorescent peak values for each stimulus from the corresponding volumes were averaged and normalized to the fluorescent peak value at the volume above the threshold level (91.7dB). Normalized fluorescent peak values remained unaltered at the LD to pharmacological manipulation (*n* = 10, 11 for the vehicle, treated respectively). **(D)** Glutamate-release at the AIS in the presence o absence of l-701 as a function of acoustic volume (dB). Normalized fluorescent peak values significantly increased at the AIS to pharmacological manipulation at volumes larger than 83.8 dB (*n* = 10, 11 for the vehicle, treated respectively, *p* < 0.01, two-sample *t*-test). No significant difference was observed at 81.8 dB. **p < 0.01, ***p < 0.001, ns, not significant.

Next, we measured escape responses and glutamate release as a function of the intensity of acoustic stimulation in the absence and presence of l-701 (Figures 3A,B). At both the LD and AIS regions, a logarithmic increase of glutamate-release was observed by increasing the stimulus intensity, with no significant difference between the two curves in control conditions (Figures 3C,D). The application of 50 μM l-701 did not alter glutamate release at the LD (Figure 3C). In contrast, NMDA receptor inhibition drastically sensitized glutamate release at the AIS region in parallel with sensitizing the escape response of fish (Figures 3B,D). Under both control and treatment conditions, escapes were elicited when glutamate release reached a certain threshold at approximately 60 percent of the maximal release, and this was regulated by NMDA receptors.

### Depression of Glutamate Release and the Modulatory Effects of NMDA Receptor Antagonism in Response to Repetitive Stimulation

**Figure 4:**
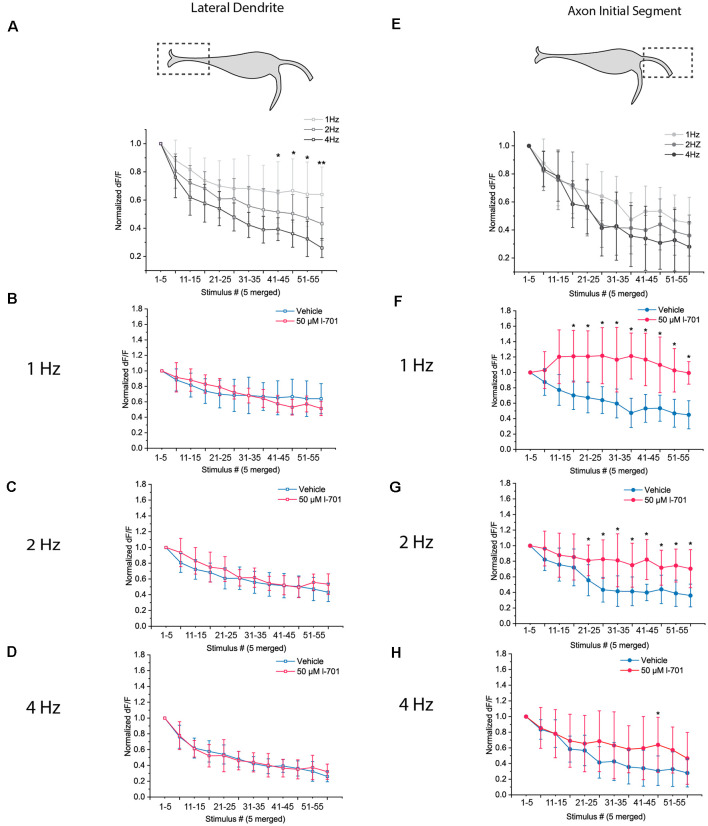
Dynamics of glutamate release in response to repetitive acoustic stimulation exhibit distinct properties at the LD and the AIS. Dynamics of presynaptic glutamate-release in response to repetitive acoustic stimulation at the regions of interest. The fish received 60 acoustic stimuli (91.7 dB) at frequencies ranging from 1 Hz to 4 Hz. Fluorescence values were recorded from both regions, peak fluorescence values of every five consecutive glutamate spike events were averaged and normalized to the mean of peak fluorescence values of the first five stimuli. Increasing the frequency of stimulation revealed different underlying mechanisms of depression in the two regions. **(A)** At the LD, depletion of presynaptic glutamate significantly increased with frequency LD [*p* values for the last four data points (stimulus 40–60) *p* = 0.03, 0.02, 0.04 and 0.002 respectively; *n* = 9, 8 and 6 for 1 Hz, 2 Hz, and 4 Hz, respectively]. **(B–D)** Bath application of 50 μM l-701 did not significantly alter the synaptic depression of glutamatergic endings at the LD at neither of the frequencies used for stimulation (two-sample *t*-test). **(E)** At the AIS, the initial rate of depression at 1 Hz could only be slightly altered with higher-frequency stimulation resulting in less significantly different data points than at the LD [*p* values for the last four data points (stimulus 40–60) *p* = 0.1, 0.06, 0.4 and 0.2, respectively; *n* = 11, 11 and 10 for 1 Hz, 2 Hz, and 4 Hz, respectively]. **(F–H)** Bath application of 50 μM l-701 significantly decreased the synaptic depression of glutamatergic nerve endings at the AIS. The perturbation was most prominent at 1 Hz (*p* < 0.05, two-sample *t*-test). The effects of 2 Hz stimulation were still significantly different from the control values (*p* < 0.05, two-sample *t*-test), whereas no significant difference was observed when comparing the values at 4 Hz stimulation (two-sample *t*-test). *p < 0.05, **p < 0.01.

Next, we sought to investigate the dynamics of glutamate release at the LD and AIS in response to repetitive acoustic stimulation. We applied sets of 60 acoustic-vibrational stimuli of different frequencies while imaging glutamate release at the two regions of interest. As stimulation above 1 Hz caused more robust habituation in our behavioral assay, we set the frequency of stimulation in the 1–4 Hz range for this experiment. Glutamate release of both subcellular regions underwent strong depression in response to repetitive stimulation, however, depression had markedly distinct properties at the two regions. The depression of glutamate release at the LD was highly dependent on the frequency of the stimuli (Figure 4A), and NMDA receptor blockade did not result in significant changes to the dynamics of depression (Figures 4B–D).

In contrast, at the AIS, an increase in the frequency of stimuli did not result in significantly different dynamics (Figure 4E). Treatment with 50 μM l-701 however resulted in drastically augmented glutamate release. The effect was most prominent at 1 Hz, where depression of glutamate release was not observed throughout the 60 stimuli (Figure 4F). At 2 Hz, the dynamics of glutamate release were still significantly different between the vehicle and treated fish (Figure 4G). At 4 Hz, no significant difference between the two groups could be observed (Figure 4H).

### Spiral Fiber Neuron Activity Alter the Startle Threshold and Habituation in an NMDA Receptor-Dependent Manner

Results obtained from imaging glutamate release at distinct subcellular regions of the M-cell revealed that inputs converging on the AIS region exhibit an NMDA receptor-dependent activity. This activity contributes to setting the startle threshold and facilitates synaptic depression during habituation. The distinct dynamics of synaptic depression at the LD and AIS regions suggested that inputs converging on the AIS could come from a feedforward excitatory cell population which plays a role in setting the startle threshold and habituation in an NMDA receptor-dependent manner.

**Figure 5:**
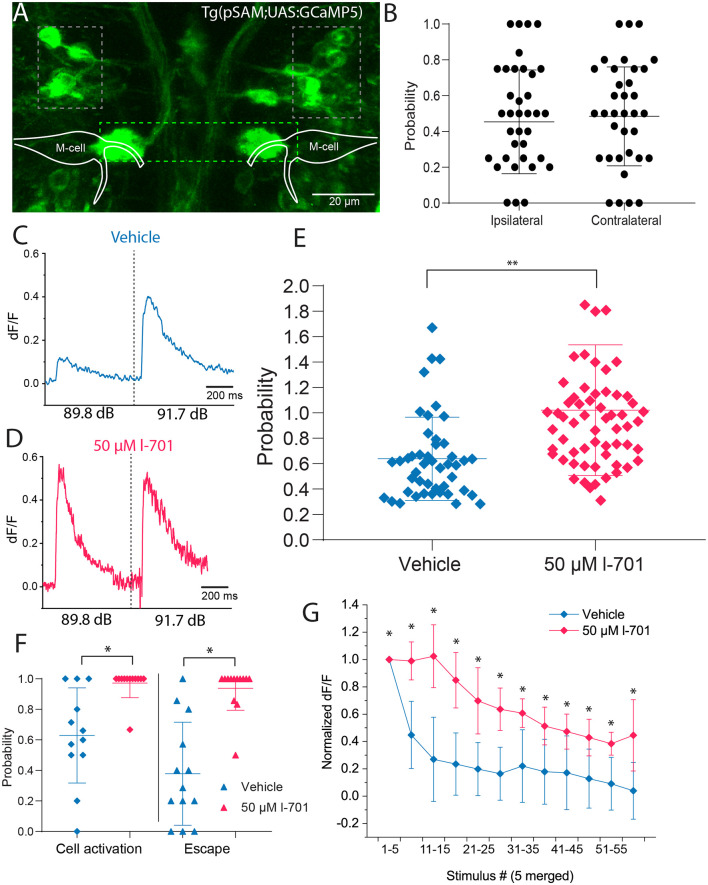
Spiral fiber neuron activity is modulated by NMDA receptor blockade in setting the startle threshold and habituation. **(A)** Representative image of the transgenic line *pSAM*. Grey boxes indicate the two clusters of SFNs. The green box shows the axon cap, a bundle of spiral fiber neuron axons surrounding the M-cell axon initial segment. **(B)** SFN activity does not significantly influence short-latency escape directionality after non-directional acoustic stimulation using our speaker system (*n* = 6 fish; five responses per fish). **(C)** Representative fluorescent trace indicating that delivering an acoustic stimulus below the startle threshold at 89.8 dB results in approximately 60% of the fluorescence level measured above the threshold at 91.7 dB. **(D)** Representative fluorescent trace indicating that administration of 50 μM l-701 increases fluorescence values at 89.8 dB to values measured at 91.7 dB. **(E)** Significant increase in fluorescence peak values in response to treatment with 50 μM l-701. Peak fluorescence values at 89.8 dB were normalized to values after stimulation at 91.7 dB (*n* = 8, 13 for vehicle, treated respectively, *p* = 0.0029, two-sample *t*-test). Lines represent the mean ± SD. **(F)** The probability of SFN activation at 89.8 dB in both vehicles and treated animals. The threshold for cell activation was defined as the mean value of baseline fluorescence of the cell +2 times the standard deviation of the baseline. At 89.8 dB control conditions, SFNs are active only 60% of the time. Treatment with l-701 results in an increase in probability of cell activation at 89.8 dB (*n* = 13 for vehicle and treated, respectively; *p* < 0.05, one-way ANOVA). **(G)** The activity of SFNs to repetitive acoustic stimulation at 1 Hz. Fluorescence peak values from every five stimuli were averaged and normalized to the mean value coming from the first five stimuli. Treatment with 50 μM l-701 significantly decreased the depression of cell activity (*n* = 6 and 8 for vehicle and treated, respectively; *p* < 0.05, two-sample *t*-test). *p < 0.05, **p < 0.01.

To test our hypothesis, we next investigated if spiral fiber neurons (SNFs), an excitatory interneuron population known to form a bundle of axons called the axon cap around the M-cell AIS (Lacoste et al., 2015), could contribute to the observed dynamics of glutamate release at the AIS. To assess the activity of SFNs, we monitored calcium levels using the *pSAM* double-transgenic zebrafish line. SFNs appear in two clusters consisting of 2–3 cells in rhombomere 3 and send axons to the AIS of the M-cells forming the so-called axon-cap, located about 10–20 μm dorsal, and 10 μm caudal to their soma (Figure 5A). We were interested in whether activities of SFNs correlated with AIS activities in setting the startle threshold and the habituation of the startle reflex.

First, we tested how the firing of SFNs correlates with the directionality of the startle response. Upon acoustic stimulation with intensities above threshold level using the speaker underneath the embedded fish, an increase in calcium levels of SFNs was observed immediately, with calcium events restricted to only a single cell in the posterior cluster of the neurons most of the time. Measuring calcium release and the directionality of the response simultaneously, results showed no significant bias of neurons firing on the contra- or ipsilateral side of the escape response, suggesting that individual cells innervate both axon cap regions surrounding the M-cell (Figure 5B).

Next, we measured the calcium activity of SFNs after a subthreshold (89.9 dB) and a strong (91.7 dB) sound pulse in the absence and presence of 50 μM l-701 (Figures 5C,D). Both the intensity and the probability of somal calcium signal were strongly dependent on the sound intensity used. NMDA receptor inhibition highly sensitized SFNs as both the intensity of the calcium signal (Figure 5E) and the probability of cell activation significantly increased at subthreshold stimulation (89.8 dB; Figure 5F). This attribute of SFNs was highly correlated with the activities observed at the AIS region of the M-cell (Figure 3D).

In the following experiment, we examined the calcium activity of SFNs during repetitive stimulation at 1 Hz. This stimulation frequency caused a massive drop in peak fluorescence even in the first few trials, with fluorescence values decreasing to 40% of their initial value by the 6th trial. This pattern showed a considerable correlation with changes in startle probability observed in our behavioral assay at this frequency (Figure 1E). NMDA receptor antagonist l-701 caused a significant increase in cell activity. SFNs under this condition maintained 80% of their initial peak fluorescence until the 20th trial (Figure 5G). This increment in the activity also correlated with the observed changes in behavior and increased glutamate release at the AIS (Figure 4F). Together, these results revealed that SFNs play an active part in not only eliciting an escape response but in setting the startle threshold and in habituation.

## Discussion

**Figure 6:**
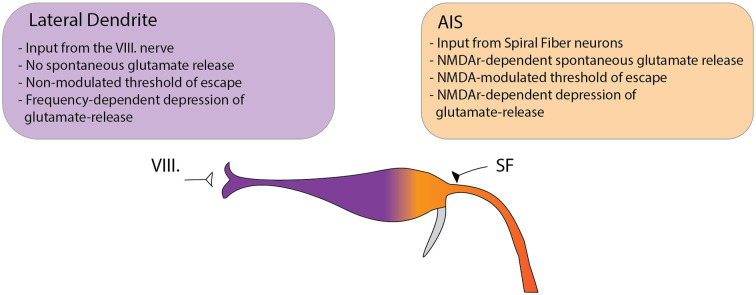
Model depicting the differential functions of glutamatergic Mauthner-cell inputs in setting the startle threshold and habituation. The M-cell receives two major glutamatergic inputs: the VIII nerve ending at the distal LD and input from SFNs ending at the proximal AIS. We observed several key differences between the two sites suggesting distinct roles in modulating the startle behavior. At the LD no spontaneous subthreshold glutamate release was observed. In setting the threshold to the startle reflex in response to acoustic stimulation, the release at the LD could not be modulated by NMDA receptor antagonism. Furthermore, the rate of depression of glutamate release to repeated stimulation was strongly dependent on the frequency of the stimuli but was not altered by NMDA receptor antagonism. In contrast, at the AIS spontaneous release events were frequently observed and could be increased by NMDA receptor blockade. In setting the startle threshold, glutamate release at the AIS strongly correlated with the probability of escape and was dependent on NMDA receptor activity. Additionally, NMDA receptor blockade strongly modulated the depression of glutamate release in response to repetitive acoustic stimulation.

Although habituation of the acoustic startle response in zebrafish has been previously suggested to be regulated by depression of synaptic activity at the LD (Marsden and Granato, 2015), the exact role of converging excitatory processes of the circuit was not fully understood. To directly assess the contribution of glutamatergic inputs in setting the startle threshold and in the regulation of habituation, we simultaneously measured glutamate release at two subcellular regions of the M-cell: the distal lateral dendrite and the proximal axon initial segment. We found that the two subcellular compartments exert strikingly distinct characteristics in setting the startle threshold and habituation (Figure 6). At the LD, spontaneous activity was absent. NMDA receptor antagonism did not alter the amplitude of glutamate release in setting the threshold for escape, nor did it affect glutamate depression during habituation. We argue that this suggests a role for the LD in setting a baseline level of excitability in the startle threshold and habituation. In stark contrast to the LD, spontaneous glutamate release events were regularly observed at the AIS. Furthermore, NMDA receptor antagonism significantly elevated glutamate release, which correlated with the increased rate of escape responses to low-volume acoustic stimulation. Additionally, a decreased level of depression was observed during habituation, again correlated with the probability of escape. Our results also unveiled that glutamate re-uptake is not modulated by NMDA receptor antagonism in these subcellular regions, because the time constants of the glutamate spikes do not change following the addition of an NMDA receptor antagonist. To unveil the origin of AIS modulation, we investigated the role of SFNs in setting the startle threshold and habituation. To this end, we imaged the calcium activity of SFNs and found that, as expected, the changes in glutamate release observed at the M-cell can be attributed to decreased sensitivity of SFNs.

Previous works exploring the roles of SFNs in the startle circuit revealed their crucial role in eliciting a proper escape response, but were viewed as a rather unregulated and hard-wired input (Lorent et al., 2001; López-Schier, 2019). Furthermore, their involvement in habituation were excluded as they converge downstream of the M-cell soma (Marsden and Granato, 2015). In contrast, our results indicate the active involvement of SFNs in adaptive processes and instead suggest a model in which the AISs serve as an interface where regulation of certain behavioral properties is achieved. The importance of SFNs and regulation at the proximal AIS is supported by the following observations: first it has been shown that excitation at the LD is attenuated by the time it reaches the soma due to the passive cable properties of the M-cell (Szabo et al., 2006). Laser ablation experiments on the M-cell show also surprising insights into the crucial role of the AIS region. A recent study has demonstrated that ablating solely the M-cell soma while leaving the AIS region intact does not result in a significant impairment in the latency of the startle response. In contrast, ablating the AIS region or the M-cell axon itself causes a significant increase in the response time of the escape (Hecker et al., 2020). One potential explanation for this is that an intact AIS region and the activation of SNFs during acoustic stimulation is sufficient to retain the properties of fast escape responses, which is further supported by the observation that SFN ablation had a more dramatic effect on the probability of startle than M-cell somatic ablations where the AIS remained largely intact (Lacoste et al., 2015). From a technical standpoint, compared to somatic calcium imaging, our experimental approach of imaging presynaptic glutamate release allows a clear distinction of subcellular activities due to finer spatial resolution, revealing previously overlooked details of M-cell activation.

The increased activity of SFN in response to NMDA receptor blockade suggests that this pathway could be innervated and regulated by inhibitory neurons. The role of feedforward inhibitory neurons in habituation of the startle response therefore could not only lie in attenuating the excitability of the M-cell which has been proposed by previous reports (Marsden and Granato, 2015), but also in the inhibition of SFN-mediated activities. The origin of this inhibition, however, remains to be elucidated, and further experiments are required to uncover the details of the whole regulatory network of the startle response.

Of note, recent evidence from the closely related cavefish (*Asytanax mexicanus*) suggests that larval rearing conditions can affect the morphology of M-cell dendrites (Tanvir et al., 2020). The larvae raised in dark up until 5 dpf have thicker dendrites than those of controls raised in 12-h light: 12-h dark conditions. We do not know yet at this point if this phenotypic plasticity occurs in zebrafish as well, but we speculate that if it occurs it makes the role of LD in escape responses even more pronounced in dark reared animals.

Regulation of a behavioral threshold by an excitatory feedforward neuron population is a motif that could provide ample computational power, filtering, and enhancement of neuronal signals; therefore, it ensures a proper and swift response to salient environmental stimuli. Several examples of such a motif have been revealed in studies, both at the population and at the single-cell level. At the population level, interactions between cooperating brain regions in certain cases show remarkable similarities to this cellular motif. For example, the hypocretin/orexin (Hcrt) neurons in mice, located exclusively in the lateral hypothalamus, play an important role in the regulation of sleep-wakefulness cycles by increasing arousal through excitatory actions (Schöne and Burdakov, 2017). These cells send axons directly to the medial prefrontal cortex (mPFC), a brain region known to be crucial in associative function and attention. Another target of Hcrt neurons is the paraventricular nucleus of the thalamus (PVT), which has very strong reciprocal excitatory connections with the mPFC. Thereby, the effect of Hcrt to promote arousal is enhanced by a circuit architecture involving both direct and indirect pathways to the mPFC (Huang et al., 2006). Another example for such an arrangement could be observed in the roles of excitatory neurons in the medial superior colliculus (mSC), and of glutamatergic neurons in the dorsal periaqueductal grey (dPAG). These brain regions perform distinct tasks in the process of computing escape decisions to abrupt stimuli in mice. Neurons of the dPAG are required for the initiation of the escape response, while the activity of mSC neurons detect the saliency of the stimulus by integrating threat evidence from different brain regions. A monosynaptic connection from the mSC to the dPAG sets up a startle threshold, which can then be overcome by high mSC network activity (Evans et al., 2018). At the single-cell level, an interesting illustration of this kind of signal processing could be found in CA1 neurons located in the hippocampus. CA1 neurons receive both monosynaptic and indirect inputs. Monosynaptic inputs converge onto distal dendrites, thus are severely attenuated when they reach the soma, whereas indirect inputs project to the proximal dendrites, providing a strong influence of overall synaptic activity (Dudman et al., 2007). It has been suggested that the distinct nature of the two types of inputs could increase computational power to the CA1 pyramidal neurons. While the proximal signals drive somatic spikes, temporally specific subthreshold signals at the distal dendrites could serve as an instructive cue for the induction of additional plasticity at proximal synapses, thereby fostering the stabilization of appropriate mnemonic information.

Even though habituation in zebrafish is viewed as a rather simple form of learning, the mechanisms of cellular and molecular components that enable the animal to adapt its behavioral state to the environment are not fully understood. Recently studies on the subject identified key regulators of habituation on the molecular level such as the GPCR CasR (Jain et al., 2018), IGFR (Wolman et al., 2015), glutamate (Tabor et al., 2018), and NF1 (Wolman et al., 2014) signaling. Furthermore, it has recently been demonstrated that molecular events restricted to well-defined subdomains of the startle circuit are essential for the modulation of habituation, such as palmitoylation of Kv1.1 channels exclusively at the lateral dendrite (Nelson et al., 2020). On the circuit level, a new population of glutamatergic neurons involved in inhibiting auditory signals at the lateral dendrite has been recently discovered (Tabor et al., 2018). Our data extend the list of observations that functional elements of the startle circuit are manifested at the subcellular level, which unveils a higher-level dynamic modulation of behavior. Leveraging from the advancements in multiphoton imaging (Griffiths et al., 2020) and neurotransmitter sensor development for GABA-A (Marvin et al., 2019) and dopamine (Sun et al., 2018), it is now within reach to study the activity dynamics of transmitter release in 3D real-time, which could reveal how animal behaviors are regulated in unprecedented detail.

## Data Availability Statement

The raw data supporting the conclusions of this article will be made available by the authors, without undue reservation.

## Ethics Statement

The animal study was reviewed and approved by Hungarian National Food Chain Safety Office (NÉBIH).

## Author Contributions

DB, MV, ÁZ, and AM-C designed the study and wrote the manuscript. IL and GS built the behavioral setup and analyzed the data. DB performed the experiments. All authors reviewed the manuscript. All authors contributed to the article and approved the submitted version.

## Conflict of Interest

IL was employed by the company Printnet Limited. AM-C was employed by the company Motorpharma Limited. The remaining authors declare that the research was conducted in the absence of any commercial or financial relationships that could be construed as a potential conflict of interest.
